# Bone quality affects stability of orthodontic miniscrews

**DOI:** 10.1038/s41598-022-06851-y

**Published:** 2022-02-18

**Authors:** Wan-Ping Yu, Ming-Tzu Tsai, Jian-Hong Yu, Heng-Li Huang, Jui-Ting Hsu

**Affiliations:** 1 Master Program for Biomedical Engineering, China Medical University, Taichung, 404 Taiwan; 2 Department of Biomedical Engineering, Hungkuang University, Taichung, 433 Taiwan; 3 School of Dentistry, College of Dentistry, China Medical University, 91 Hsueh-Shih Road, Taichung, 40402 Taiwan; 4 Department of Dentistry, China Medical University and Hospital, Taichung, 404 Taiwan; 5 Department of Bioinformatics and Medical Engineering, Asia University, Taichung, 413 Taiwan

**Keywords:** Anatomy, Diseases, Health care, Medical research, Physics

## Abstract

The objective of this study was to evaluate the effect of bone–miniscrew contact percentage (BMC%) and bone quality and quantity on orthodontic miniscrew stability and the maximum insertion torque value (ITV). Orthodontic miniscrews of five different dimensions and several bovine iliac bone specimens were used in the evaluation. Miniscrews of each dimension group were inserted into 20 positions in bovine iliac bone specimens. The experiment was divided into three parts: (1) Bone quality and quantity were evaluated using cone-beam computed tomography (CBCT) and microcomputed tomography. (2) The 3D BMC% was calculated. (3) The ITVs during miniscrew insertion were recorded to evaluate the stability of the orthodontic miniscrews. The results indicated that longer and thicker miniscrews enabled higher ITVs. CBCT was used to accurately measure cortical bone thickness (r = 0.939, *P* < 0.05) and to predict the bone volume fraction of cancellous bone (r = 0.752, *P* < 0.05). BMC% was significantly influenced by miniscrew length. The contribution of cortical bone thickness to the ITV is greater than that of cancellous bone structure, and the contribution of cortical bone thickness to BMC% is greater than that of cancellous bone structure. Finally, the higher is BMC%, the greater is the ITV. This study concludes that use of CBCT may predict the mechanical stability of orthodontic miniscrews.

## Introduction

Orthodontic treatment involves improving and adjusting misaligned teeth using fixed or movable orthodontic devices. To move such teeth more effectively, a fixed point is usually selected as an anchor point for pulling teeth; therefore, anchorage control plays an important role in orthodontic treatment^1–4^. Related treatment usually involves using the posterior molar as an anchorage point, springs are applied to brackets to pull anterior teeth backward. However, excessive spring tension may produce a reaction force on anchor teeth, causing the patient to feel uncomfortable during treatment or loosening anchor teeth^5,6^. To overcome this disadvantage, orthodontic miniscrews have been developed recently. An orthodontic miniscrew is inserted between the root of two teeth to provide strong and fixed anchorage. The use of orthodontic miniscrews has various advantages, including easy insertion and removal, a multitude of insertion position options, simple surgical procedures, smaller surgical wounds, a short wound recovery time, and low price^7–10^.

Despite the aforementioned advantages, surgical failure may arise, such as accidental injury to the surrounding tissue (root of the tooth and inferior alveolar nerve), inflammation and infection of surrounding tissue after insertion, and the fracturing of orthodontic miniscrew caused by excessive torque during removal. However, the main reason behind such failure may be that insertion positions cannot provide sufficient stability for the orthodontic miniscrew^11–13^. The lower is the stability after inserting, the higher is the possibility of orthodontic miniscrew loosening^14,15^. In clinical practice and research, many methods are available to evaluate orthodontic miniscrew stability; among these methods, the most widely used is maximum insertion torque value (ITV) measurement during insertion. The maximum ITV is often used as an index to evaluate orthodontic miniscrew stability^16^.

Many factors affect the stability of miniscrews, including exterior design and the insertion method adopted by the attending dentist. However, the most important factors affecting stability are the cortical bone thickness and the cancellous bone structure at the insertion position of the patient’s jaw bones^17^. In clinical, the quality and quantity of jaw bones at the orthodontic miniscrew insertion position are often evaluated using two dimensional (2D) and three dimensional (3D) images. Compared with 2D images, 3D images are less likely to encounter problems such as image distortion, and images can be reconstructed on a computer to obtain internal structural images of the position to be measured. Therefore, 3D images are often used as an auxiliary tool for clinical quantitative measurement of maxilla and mandible bone quality^18,19^. Britz et al.^20^ have indicated that the images obtained from microcomputed tomography (micro-CT) are the gold standard for evaluating trabecular bone microstructure. However, micro-CT images are not useful in clinical examinations due to the small scanning range of micro-CT. Recently, cone-beam computed tomography (CBCT) has often been used to capture 3D images in clinical dentistry. However, most related studies that have used CBCT to evaluate orthodontic miniscrew stability have focused on the influence of cortical bone thickness at the insertion position on orthodontic miniscrew stability^21^; studies that have focused on cancellous bone density are relatively rare. Several researchers have used CBCT to evaluate the bone quality and mass of the jawbone at the dental implant site^22–24^. In addition, numerous researchers have discussed the influence of bone implant contact (BIC) level on dental implant stability after insertion. However, most studies have focused on dental implants^25–27^; studies that have explored the correlation between orthodontic miniscrew stability after insertion and the bone–miniscrew contact percentage (BMC%) are relatively rare^28,29^.

Studies related to orthodontic miniscrews have mostly explored the effect of different miniscrew brands or exterior designs on orthodontic miniscrew stability^30–34^; whether bone quality or quantity influence stability is less discussed^29^. Furthermore, no study has examined the correlations between the three factors that affect orthodontic miniscrew stability, namely bone quality and quantity, BMC%, and the ITV. Therefore, the aim of this study was to predict bone structure at the orthodontic miniscrew insertion position using CBCT and determined the influence of orthodontic miniscrew on mechanical stability after insertion.

## Results

### The effect of miniscrew dimension on ITV

In the experiment, orthodontic miniscrews of five different dimensions were used, with 20 orthodontic miniscrews in each group and 100 specimens in total. Table 1 shows the maximum ITV of the five orthodontic miniscrews inserted into the bone specimen; dimension appeared to significantly influence the maximum ITV (P < 0.05). The specifications were grouped for comparison according to length and diameter: First, no statistical difference (P = 0.091) was noted in the influence of orthodontic miniscrews with the same diameter (1.5 mm) and different lengths (6, 8, and 10 mm) on the maximum ITV; however, the longer was the miniscrew, the higher was the maximum ITV. Orthodontic miniscrews with different diameters (1.4, 1.5, and 1.6 mm) and the same length (8 mm) did not have significantly different effects on the maximum ITV (P = 0.07); however, the greater was the diameter, the greater was the maximum ITV. The post hoc Mann Whitney *U* test found that the miniscrew groups of different dimensions did not different significantly (1.4 × 8 mm miniscrew group and 1.5 × 8 mm miniscrew group: P = 0.417; 1.5 × 8 mm miniscrew group and 1.6 × 8 mm miniscrew group: P = 0.113; 1.5 × 6 mm miniscrew group and 1.5 × 8 mm miniscrew group: P = 0.208; 1.5 × 8 mm miniscrew group and 1.5 × 10 mm miniscrew group: P = 0.199), but the dimension differences were significantly different (1.4 × 8 mm miniscrew group and 1.6 × 8 mm miniscrew group: P < 0.05; 1.5 × 6 mm miniscrew group and 1.5 × 10 mm miniscrew group : P < 0.05).

**Table 1:** ITVs based on different orthodontic miniscrew dimensions.

Miniscrew dimension	Diameter × length (mm)					P value
	1.4 × 8	1.5 × 6	1.5 × 8	1.5 × 10	1.6 × 8	
ITV median	13.7	12.7	15.3	15.4	18.6	< 0.05
IQR	8.5	7.1	11.0	10.0	0.4	
Max	20.6	19.1	22.7	26.9	29.8	
Min	4.0	3.8	5.3	4.6	7.1	

*IQR* interquartile range, *Max* maximum, *Min* minimum.

### CBCT/Micro-CT scanning and bone quality and quantity measurement

#### Relationship between cortical bone thickness measured from CBCT and micro-CT

**Figure 1:**
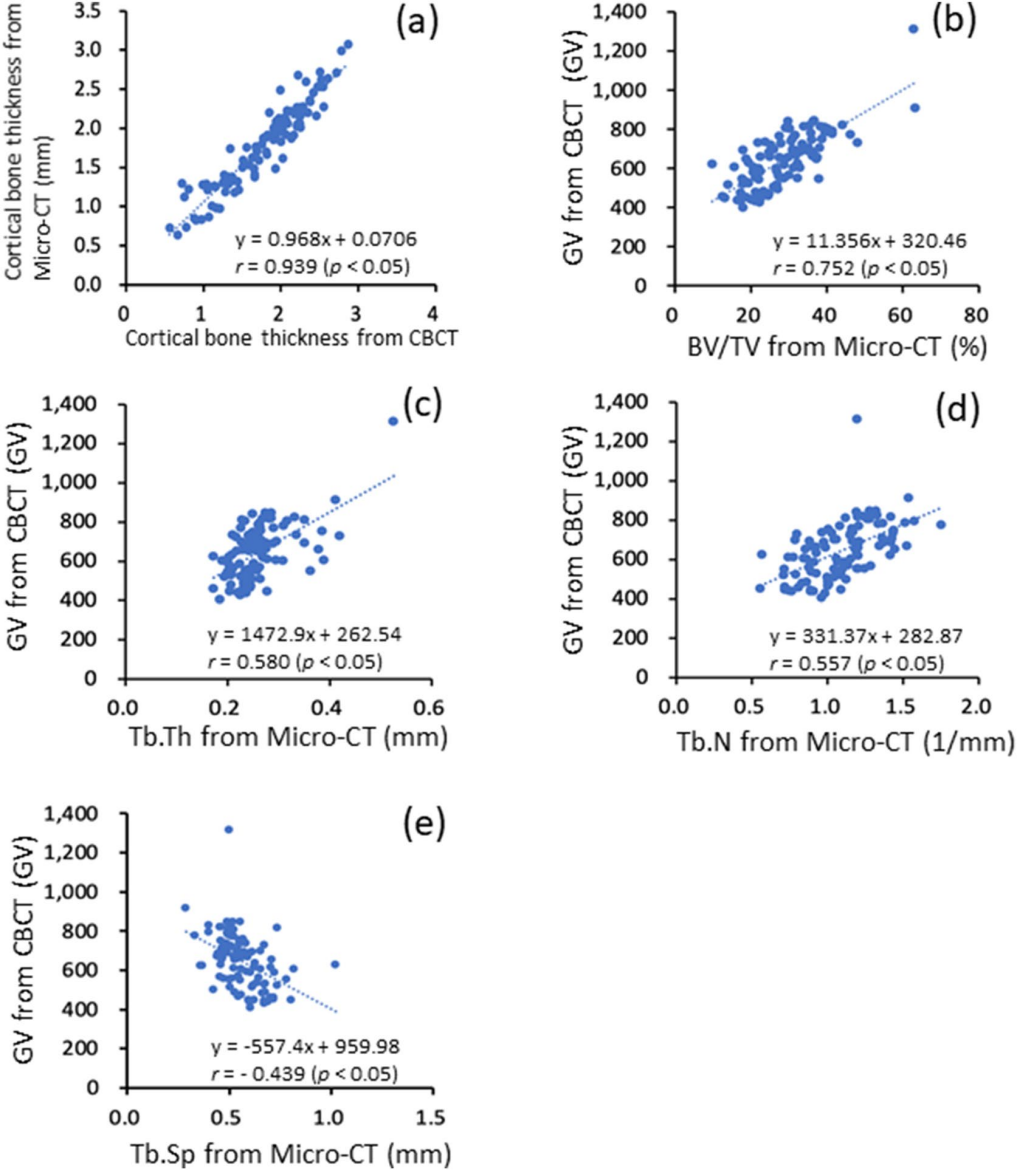
(**a**) Relationship between cortical bone thickness measured from CBCT and micro-CT images. (**b**–**e**) Relationship between cancellous bone density measured from CBCT images and four trabecular bone microstructural parameters measured from micro-CT images.

This study compared cortical bone thickness measured using the two instruments through a paired t-test (P value = 0.389). The results indicated cortical bone thickness measured using different imaging techniques was the same, and a highly positive correlation between them was noted (r = 0.939, P value < 0.05; Fig. 1a).

#### Relationship between GV measured from CBCT and four trabecular bone microstructure parameters measured from micro-CT

A high correlation was observed between measured GV and BV/TV (r = 0.752, P value < 0.05), a moderate correlation was observed between measured GV and Tb.Th (r = 0.580, P value < 0.05), a moderate correlation was observed between measured GV and Tb.N (r = 0.557, P value < 0.05), and a moderate correlation was observed between measured GV and Tb.Sp (r = − 0.439, P < 0.05; Fig. 1b–e).

### Effect of miniscrew dimension on BMC%

Table 2 shows the BMC% of five orthodontic miniscrews. Dimension had a significant effect on BMC% (P < 0.05). The specimens were grouped and compared according to their length and diameter: under the same diameter condition (1.5 mm), the length (6, 8, and 10 mm) of orthodontic miniscrews had a significant effect on BMC% (P < 0.05), and the Mann Whitney *U* test revealed statistically significant differences (P < 0.05); the shorter was the length, the higher was the BMC%. Under the same length condition (8 mm), orthodontic miniscrew diameter had no significant influence on BMC% (P = 0.718).

**Table 2:** BMC% among five different miniscrew dimensions.

Miniscrew dimension	Diameter × length (mm)					P value
	1.4 × 8	1.5 × 6	1.5 × 8	1.5 × 10	1.6 × 8	
BMC% median	46.44	61.78	48.28	38.40	48.79	< 0.05
IQR	14.66	18.34	16.20	13.02	0.90	
Max	68.00	78.58	61.06	51.76	62.44	
Min	32.66	39.85	32.33	25.13	29.65	

### Relationship between ITV and bone quality and quantity

**Figure 2:**
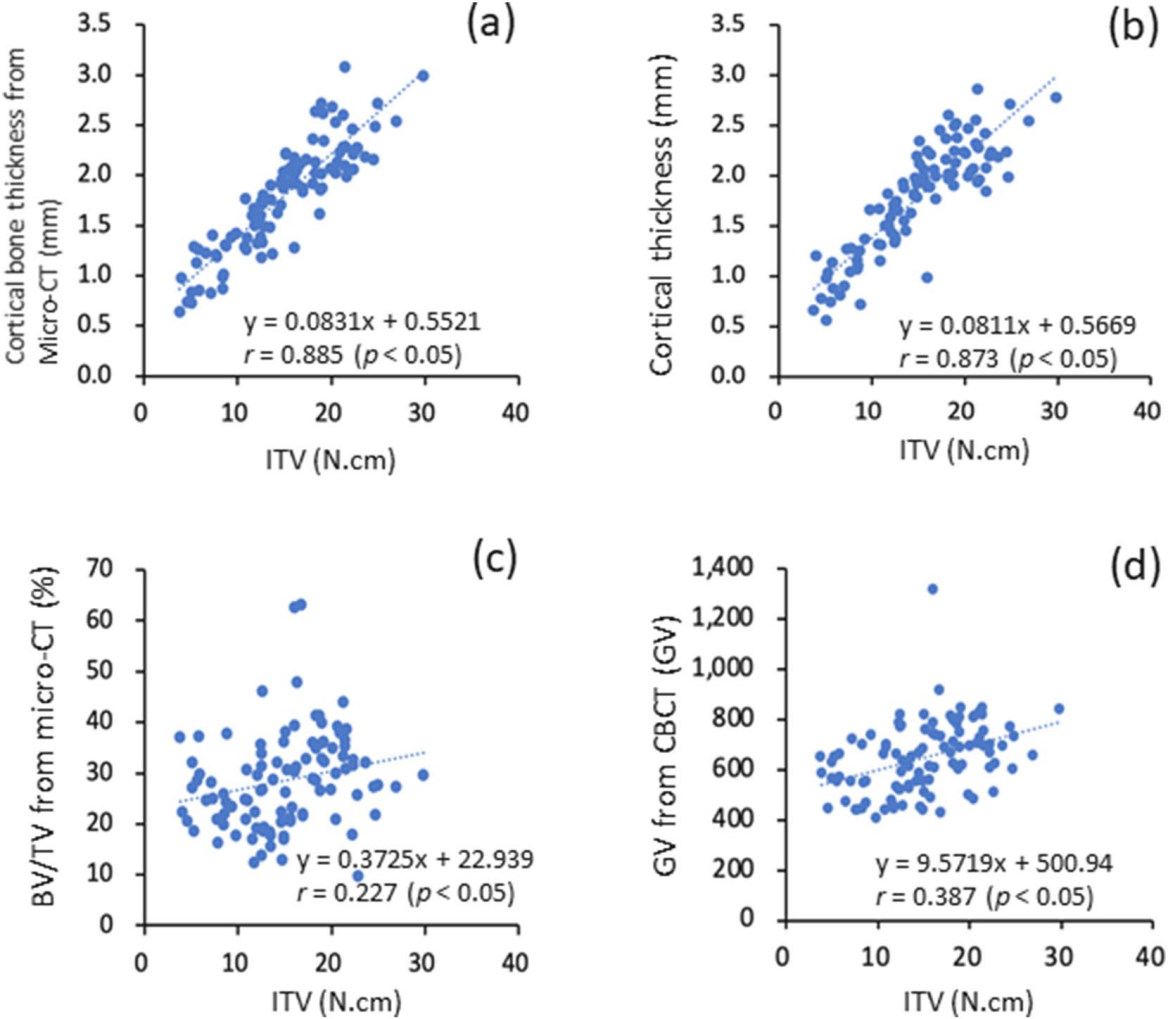
Relationships between ITV and bone quality and quantity parameters: (**a**) cortical bone thickness measured from micro-CT images, (**b**) cortical bone thickness measured from CBCT images, (**c**) BV/TV measured from micro-CT images, and (**d**) GV measured from CBCT images.

The results indicated that the maximum ITV and cortical bone thickness measured using micro-CT images were highly correlated (r = 0.885, P < 0.05; Fig. 2a). Similarly, a high correlation existed between the maximum ITV and cortical bone thickness measured using CBCT images (r = 0.873, P < 0.05; Fig. 2b). A low correlation was observed between cortical bone thickness and trabecular bone microstructure BV/TV measured using micro-CT images (r = 0.227, P < 0.05; Fig. 2c.). Finally, a low correlation was observed between the maximum ITV and cancellous bone density measured using CBCT images (r = 0.387, P < 0.05; Fig. 2d).

### Relationship between BMC% and bone quality and quantity

This section details the correlation between (1) BMC% and cortical bone thickness and (2) BMC% and cancellous bone density/trabecular bone microstructure. BMC% and cortical bone thickness in all miniscrew groups were highly correlated (r = 0.775–0.878). The correlation between BMC% and cancellous bone density/trabecular bone microstructure can be observed in Table 3. BMC% and cancellous bone density/trabecular bone microstructure of most orthodontic miniscrew groups were moderately to highly correlated (r = 0.577–0.776). The only exceptions are as follows: BMC% and the cancellous bone density of the 1.5 × 6 mm miniscrew group were moderately correlated (r = 0.577), and nonsignificant correlation was found between BMC% and the trabecular bone microstructure of the 1.5 × 6 mm miniscrew group (r = 0.373, P = 0.105).

**Table 3:** Relationship between BMC% and bone quality for different miniscrew dimensions.

Bone quality and quantity parameters	Dimension of miniscrew (diameter × length in mm)				
	1.4 × 8	1.5 × 6	1.5 × 8	1.5 × 10	1.6 × 8
Cortical bone thickness from CBCT	0.830	0.802	0.855	0.843	0.828
Cortical bone thickness from micro-CT	0.871	0.856	0.775	0.878	0.877
GV from CBCT	0.694	0.577	0.608	0.702	0.776
BV/TV from Micro-CT	0.696	0.373	0.662	0.584	0.662

Except for the relationship between BV/TV measured from micro-CT and BMC% in the 1.5 × 6 mm group, all relationships had P values less than 0.05.

### Relationship between BMC% and ITV

**Figure 3:**
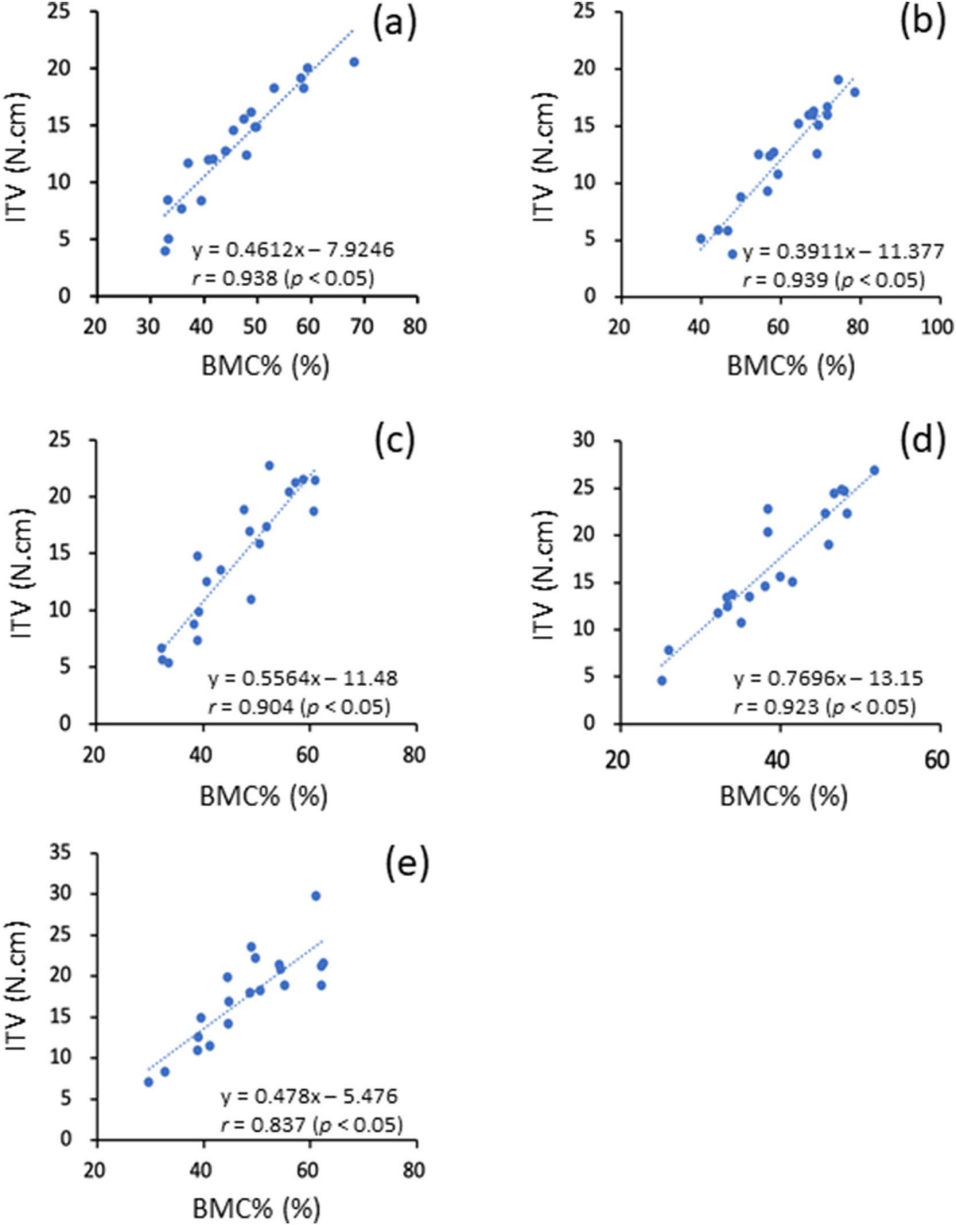
Relationships between ITV and BMC% in the (**a**) 1.4 × 8 mm^2^ miniscrew group, (**b**) 1.5 × 6 mm^2^ miniscrew group, (**c**) 1.5 × 8 mm^2^ miniscrew group, (**d**) 1.5 × 10 mm^2^ miniscrew group, (**e**) and 1.6 × 8 mm^2^ miniscrew group.

A high correlation existed between the maximum ITV and BMC% of the each orthodontic miniscrew group. The r value for the correlation between the ITV and BMC% of the 1.4 × 8 mm miniscrew group was 0.938 (P < 0.05; Fig. 3a); the r value for correlation between the ITV and BMC% of the 1.5 × 6 mm miniscrew group was 0.939 (P < 0.05; Fig. 3b); the r value for the correlation between the ITV and BMC% of the 1.5 × 8 mm miniscrew group was 0.904 (P < 0.05; Fig. 3c); the r value for the correlation between the ITV and BMC% of the 1.5 × 10 mm miniscrew group was 0.923 (P value < 0.05; Fig. 3d); and r value for the correlation between the ITV and BMC% of the 1.6 × 8 mm miniscrew group was 0.837 (P < 0.05; Fig. 3e)

## Discussion

Orthodontic miniscrews have been widely used in orthodontic treatment. However, most studies on orthodontic miniscrews have compared the effects of orthodontic miniscrew design on stability^30–32,34^; few have discussed bone structure in the insertion position^21,29,35^. In addition, no study has discussed the relationships between bone quality and quantity, BMC%, and orthodontic miniscrew stability. Therefore, in this study, orthodontic miniscrews were inserted into bone specimens, and the correlations between maximum ITV, bone quality and quantity, and BMC% were evaluated. The researchers used this approach to assess methods of predicting the orthodontic miniscrew stability using bone quality and quantity according to CBCT images to gain a better understanding of bone structure in the insertion position. This could increase the success rate of orthodontic miniscrew surgery.

Many studies have indicated that orthodontic miniscrew stability is a key factors affecting the success of orthodontic miniscrew surgery^11–15^. Other studies have indicated that the factors affecting the stability of orthodontic miniscrews after insertion include exterior design of the orthodontic miniscrew, insertion modalities adopted by the dentist, and bone quality and quantity^17^. Orthodontic miniscrew stability is closely related to the smooth progression of orthodontic treatment. The lower stability is after insertion, the greater is the risk that an orthodontic miniscrew will loosen and become detached from its position. Many methods can be used to evaluate orthodontic miniscrew stability, such as the measurement of the maximum ITV and Periotest value in mechanical experiments^36,37^, the measurement of bone quality and quantity, and determination of the level of bone-miniscrew contact in image-based measurements. Many studies have used the maximum ITV during the insertion process as an index to evaluate orthodontic miniscrew stability^16,38,39^. The Periotest is an electronic device originally used to measure changes in the damping of periodontal ligament tissue. The current generation of hand-held periodontal testers is mostly used to test bone hardness around artificial root implants and determine implant stability, but researchers such devices to test orthodontic miniscrew stability. Given that the head of the orthodontic miniscrew used in this study is small, this experiment did not consider using a Periotest to measure orthodontic miniscrew stability.

In previous studies examining orthodontic miniscrews, various bone specimens have been used, including human cadaver bones^40,41^, artificial bones^15,29^, and animal bones^42–44^. Fresh human cadaver bones are costly and difficult to obtain. Although artificial bones offer advantages such as easy determination of specimen material properties and increased convenience in experiments, they have different properties than real bones. Therefore, this experiment used animal bones. In previous studies, porcine ribs, porcine femoral heads, and bovine pelvises were used as bone specimen materials^45–47^. Some studies have indicated that bovine pelvis bone is closest to that of human maxilla and mandible bones^42^. Therefore, bovine pelvis bones were selected as the bone specimen in this study. In the clinical study of Watanabe et al.^48^, 1.4 × 6 mm miniscrews were inserted into the maxilla of 60 women with an average age of 25.4 years. The maximum ITV range measured in the study was 8.5 ± 2.1 N.cm. Suzuki et al.^49^ inserted 1.5 × 6 mm orthodontic miniscrews or 1.5 × 8 mm orthodontic miniscrews into the maxilla and mandible of 105 patients (30 male and 75 female patients) with a mean age of 20.9 years. The maximum ITV range measured in that study was between 13.6 and 15.3 Ncm. The maximum ITV range measured in this study was consistent with these results. Therefore, bovine iliac bone is acceptable as for use as a bone specimen.

In a comparison of different orthodontic miniscrew dimensions on the impact of maximum ITV, Lim et al.^48^ compared the maximum ITV of cylindrical and tapered orthodontic miniscrews with different lengths. The results indicated that the greater was the miniscrew length, the greater was the maximum ITV. However, only the cylindrical orthodontic miniscrews have significant effects on the maximum ITV (P = 0.021). Lim et al.^48^ also discussed the effect of cylindrical orthodontic miniscrews with the same length but different diameters on maximum ITV. The difference between the diameters of the selected miniscrews was approximately 3 mm, which is statistically significant (P < 0.001). Möhlhenrich et al.^49^ compared the effects of different dimensions of orthodontic miniscrews inserted into artificial bones with different bone quality on the maximum ITV. The results showed that the different bone quality of artificial bones affected the maximum ITV. The five miniscrew types and dimensions used in this study were those commonly used in clinical orthodontic treatment. However, the dimension differences in orthodontic miniscrew were small, resulting in no significant differences being evident in the maximum ITV between the groups of orthodontic miniscrews with different dimensions (1.4 × 8 mm miniscrew group and 1.5 × 8 mm miniscrew group: P = 0.417; 1.5 × 8 mm miniscrew group and 1.6 × 8 mm miniscrew group: P = 0.113; 1.5 × 6 mm miniscrew group and 1.5 × 8 mm miniscrew group: P = 0.208; 1.5 × 8 mm miniscrew group and 1.5 × 10 mm miniscrew group: P = 0.199). However, the bigger was the diameter or the greater was the length of the orthodontic miniscrew, the higher was the maximum ITV. All The aforementioned studies and the experimental results have indicated that the maximum ITV was affected by orthodontic miniscrew dimensions and differed according to the different designs of orthodontic miniscrews and the different bone quality in which the miniscrews were inserted.

The trabecular bone microstructure of cancellous bone is one of the crucial factors in evaluating bone quality and quantity, and the evaluation of trabecular bone microstructure is highly dependent on the scanning resolution of the imaging technology^50^. The micro-CT image is the gold standard for the evaluation of trabecular bone microstructure. However, micro-CT scanning cannot be used in clinical examinations due to its small scanning range. With the increasing development of the techniques of CBCT, it has come to be widely used in clinical dental examinations. Some researchers previously indicated that CBCT was not suitable for determining bone density^51,52^. However, more researchers have since demonstrated that the use of CBCT is a reliable approach for measuring bone density^24,53–56^. Suttapreyasri et al.^57^ compared cortical bone thickness measured using CBCT and micro-CT. The results indicated no difference between cortical bone thickness measured using the two imaging modalities, indicating a high correlation between CBCT and micro-CT measurements (r = 0.993, P < 0.01). In this study, paired t tests were used to compare cortical bone thickness measurements from CBCT with those from micro-CT images. The P value was 0.389, and a highly positive correlation was noted between the measurements (r = 0.939, P < 0.05). Tsutusmi et al.^58^ reported that when the cortical bone thickness was 3 to 4 times greater than the CBCT scanning voxel resolution, CBCT is suitable for measuring cortical bone thickness of the bone specimens. In this study, the CBCT scanning resolution was 200 μm, and the cortical bone thickness was greater than the scanning resolution by 3 to 4 times. Tsutsumi et al.^58^ and Hsu et al.^59^ have also compared the correlation between bone mineral density (BMD) and the aforementioned four trabecular bone microstructural parameters measured using micro-CT. The results indicated that BMD was highly correlated with BV/TV but had only a low to moderate correlation the other three trabecular bone microstructures. The results of this study are consistent those of most related studies.

Research on the degree of bone–implant contact has mostly focused on the correlations between BIC% and the stability of dental implants after insertion. Two methods are available for evaluating BIC%, namely 2D BIC% and 3D BIC%. Zhou et al.^27^ revealed a highly positive correlation between implant stability ISQ and 2D BIC% (r = 0.646). However, some studies have reported no correlation between ISQ and 2D BIC%^60,61^. One possible reason for this result is that only one 2D tissue slice was used—a single 2D tissue slice cannot fully show 3D bone and implant contact sites. Rebaudi et al.^62^ used micro-CT to measure 3D bone–implant contact and indicated that micro-CT was suitable for evaluating the degree of bone–implant contact. A previous study by our team^63^ demonstrated a significant difference between dental implants of varying lengths and 3D BIC% measured using micro-CT images (*P* < 0.05). Studies have investigated bone–miniscrew contact levels and stability after insertion. Inaba et al.^28^ inserted orthodontic miniscrews into the jawbones of adult rabbits and determined 2D BMC% using an electron microscope; BMC% in this study was 54.48 ± 11.74. In a previous study by our team^29^, we captured micro-CT images of orthodontic miniscrews and four types of artificial bone with different bone strengths, imported these images into image software, and calculate 3D BMC% using a Boolean operation. The BMC% range was 18–43. BMC% in this research fell within the values of two aforementioned studies. Furthermore, significant differences were noted between the five dimensions of orthodontic miniscrews and BMC% (P < 0.05). Significant differences were noted in the effects of miniscrews of the same diameter (1.5 mm) but different lengths (6, 8, and 10 mm) on BMC% (P value < 0.05). In the post hoc Mann Whitney *U* test, significant differences were noted in paired comparison (P < 0.05); the shorter was the miniscrew length, the greater was the BMC% value. This is because when the miniscrew is short, a greater portion of it enters the cortical bone. The same length (8 mm), but different diameter (1.4, 1.5, and 1.6 mm) of orthodontic miniscrews had no effect on BMC% (P = 0.718). In a post hoc Mann Whitney *U* test, nonsignificant differences were noted in paired comparison (P > 0.05), which may be due to the small difference in the diameter of orthodontic miniscrews selected in this study. Because the range of orthodontic miniscrew diameters used in clinical settings is limited, diameter is inferred to exert no effect on BMC%.

Regarding studies on the correlation between the maximum ITV and bone quality and quantity, Marquezan et al.^21,45^ divided bovine pelvis specimens into four groups after relevant processing according to bone region and the presence of cortical bone: the four groups were iliac bone regions without cortical bones (GI0), iliac bone regions with cortical bones (GI1), pubic bone regions without cortical bones (GP0), and pubic bone regions with cortical bones (GP1). The results indicated that the BMD of the test specimens at GP0 was larger than that of the test specimens at GI1. However, the maximum ITV required for the test specimens at GP0 is smaller than that for those at GI1. In other words, although the BMD of the bone specimens from the region with no cortical bones was larger than that of the bone specimens from a region with cortical bones, the bone specimens from the regions with cortical bone require a larger maximum ITV. This indicated that cortical bones exert a greater influence on maximum ITV than do cancellous bones. Similar results were also obtained in the current study: a high correlation exists between the maximum ITV and cortical bone thickness measured using CBCT and micro-CT. This indicated that the thicker is cortical bone, the higher the level of insertion torque value required for insertion. The GV of cancellous bone measured by CBCT and the trabecular bone microstructure parameter BV/TV measured by micro-CT had only a low to medium correlation with the maximum ITV. Therefore, the contribution of cortical bone thickness to the maximum ITV was greater than that of cancellous bone density.

Regarding the correlation between BMC% and bone quality and quantity at the insertion position, the results of this study indicated that the BMC% value of the orthodontic miniscrew of each dimension is highly correlated with cortical bone thickness (r > 0.8). The correlation results for BMC% and cancellous bone density/trabecular bone microstructure presented in Table 3 indicate that the BMC% of most orthodontic miniscrew groups was moderately to highly correlated with cancellous bone density/trabecular bone microstructure. However, the BMC% and cancellous bone density of the 1.5 × 6 mm miniscrew group were moderately correlated (r = 0.577, P < 0.05). No significant correlation was found between the BMC% value and trabecular bone microstructure of the 1.5 × 6 mm miniscrew group (r = 0.373, P = 0.105). The possible reason behind this is that the orthodontic miniscrews in this group were too short and the proportion of miniscrews in the cancellous bone was too small. Therefore, cortical bone thickness exerts a marked influence on BMC%. To determine the relationship between BMC% and cancellous bone structure, the length of orthodontic miniscrews must be taken into consideration. The shorter is the miniscrew, the smaller is the proportion of the miniscrew present in the cancellous bone and the smaller is the impact of these factors on BMC%.

Regarding correlations between BMC% and the maximum ITV, Inaba et al.^28^ inserted 1.4 × 4 mm miniscrews into the jawbone of adult rabbits and compared the correlation between BMC% and Periotest stability; the results indicated a high correlation (r = 0.722) between the two variables. Our team previously^29^ inserted 1.6 × 10 mm miniscrews into artificial bone specimens and compared the correlation between BMC% and the maximum ITV. The results indicated a high correlation between the two (r = 0.97). The aforementioned studies have only used orthodontic miniscrews of a single dimension. In this study, we used five dimensions of orthodontic miniscrews for further evaluation. The results revealed a high correlation between maximum ITV and BMC% of each different orthodontic miniscrew dimensions group. The r value for the correlation between a group of 1.4 × 8 mm orthodontic miniscrews was 0.938 (P < 0.05); the r value for the correlation between a group of 1.5 × 6 mm orthodontic miniscrews was 0.939 (P < 0.05); the same value for the correlation between a group of 1.5 × 8 mm orthodontic miniscrews was 0.904 (P < 0.05); the r value for the correlation between a group of 1.5 × 10 mm orthodontic miniscrews was 0.923 (P < 0.05); and the r value for the correlation between a group of 1.6 × 8 mm orthodontic miniscrews was 0.837 (P < 0.05). Therefore, BMC% is a useful measure for evaluating the stability of orthodontic miniscrews. The higher BMC% is, the higher is the maximum ITV for inserting orthodontic miniscrews into a bone specimens.

The limitations of this study are as follows. Consistent with the protocols used in previous studies, the bone specimens used in this study were obtained from animals. In the future, animal bone specimens from a greater variety of animal species or human bone specimens should be used to confirm the findings of this study. Only five representative dimensions of orthodontic miniscrew were selected for stability evaluation. The effect of other dimensions on the stability of orthodontic miniscrews still requires investigation. Furthermore, an in vitro mechanical experiment cannot simulate actual situations of clinical dentists inserting orthodontic miniscrews into a patient’s jawbone. Finally, this study only examined insertion torque value, bone quality and quantity, and the stability of bone–miniscrew contact. It did not discuss inflammation, infection, fracture during removal, or other concerns.

In conclusion, Under the experimental configuration and limitations, the following findings were obtained:

1. CBCT can be used to accurately measure cortical bone thickness and predict the BV/TV value of cancellous bone.
2. BMC% is significantly affected by orthodontic miniscrew length.
3. The contribution of cortical bone thickness to maximum ITV is greater than that of cancellous bone structure.
4. The contribution of cortical bone thickness to BMC% is greater than that of cancellous bone structure
5. The higher is BMC%, the larger is the maximum ITV.

## Materials and methods

**Figure 4:**
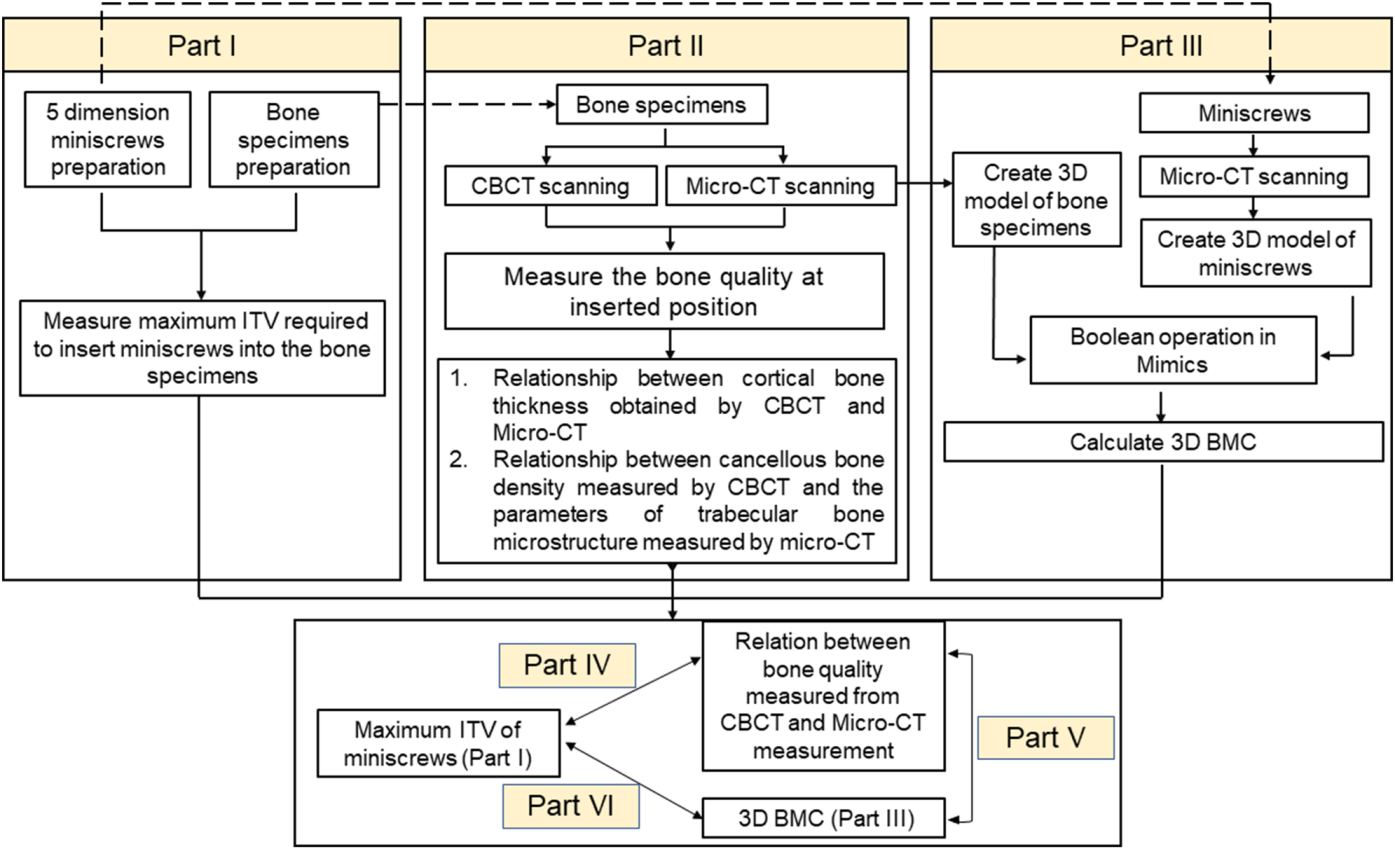
Outline of the study.

The prospective study was performed according to the STARD guidelines. The study design is illustrated in Fig. 4. In this study, 9 parameters in total were measured or calculated. As reported in the 2nd subsection of the “Materials and Methods” section, cortical bone thickness and the cancellous bone grayscale values for the inserted positions of the orthodontic miniscrews in CBCT images were measured, and the cortical bone thickness and four trabecular bone structure parameters of the inserted position of the orthodontic miniscrews in the micro-CT images were measured. As described in the 3rd subsection of the Materials and Methods section, the BMC% parameters were calculated, and as explained in the 4th subsection, the maximum ITVs were recorded.

### Bone specimen and orthodontic miniscrew preparation

**Figure 5:**
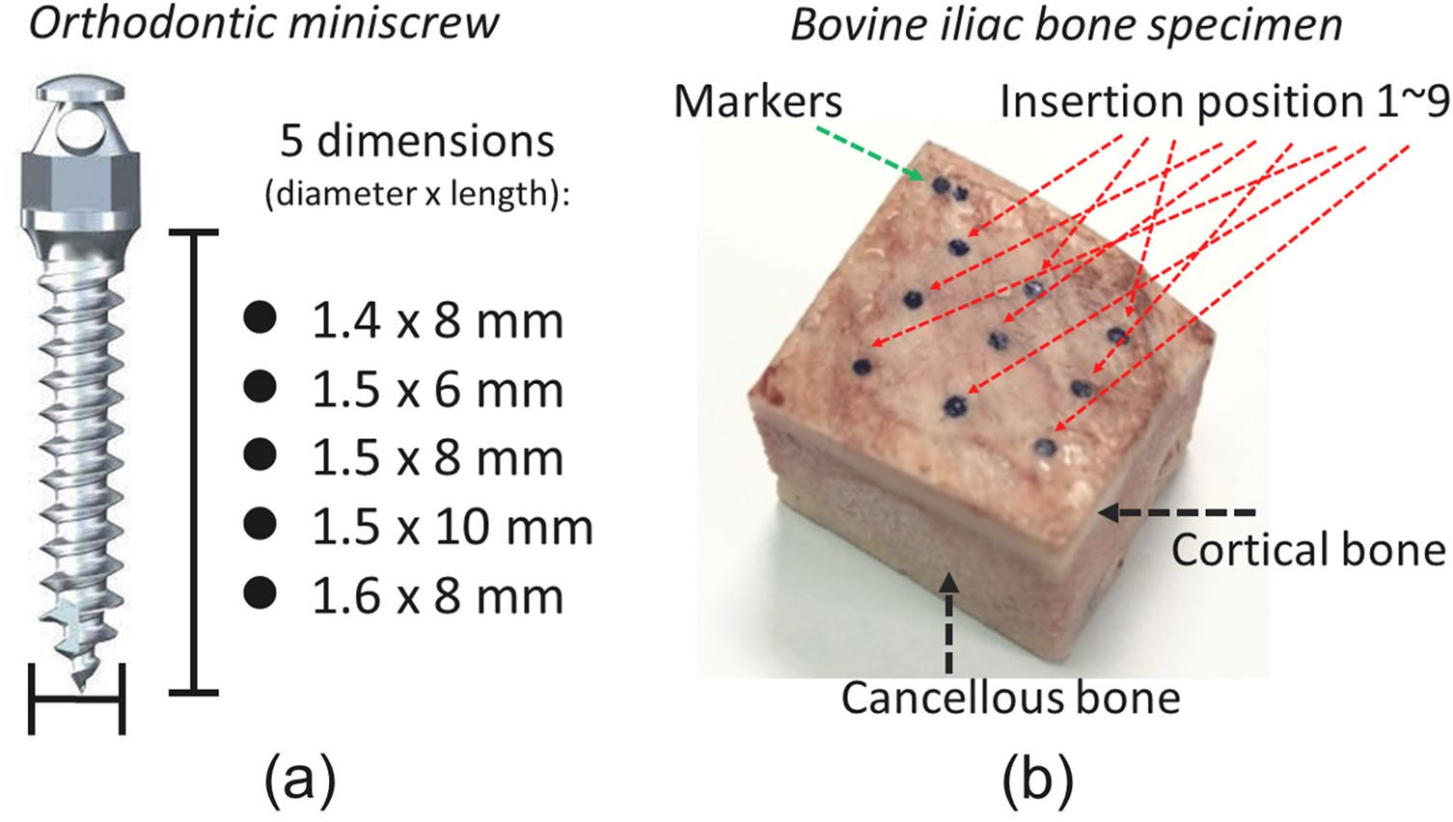
(**a**) Dimensions of orthodontic miniscrews; (**b**) Bovine iliac bone specimen showing nine insertion positions.

Twelve bone specimens were extracted from the iliac region of four bovine pelvises using an electric chainsaw. The fresh frozen bovine bone specimens were obtained from a local meat market. The dimension of each bone specimen was approximately 3 × 3 × 2 cm^3^, and two small holes were drilled on a corner of bone specimens as markers; the markers served as a reference for relative corresponding positions in images and actual mechanical experiments. In bone specimen, nine points were marked as the positions to be inserted with miniscrews (Fig. 5a). Absoanchor orthodontic miniscrews of five different dimensions (Dentos, Daegu, South Korea) were used. The dimensions of the five miniscrew were as follows: 1.4 mm × 8 mm, 1.5 mm × 6 mm, 1.5 mm × 8 mm, 1.5 mm × 10 mm, and 1.6 mm × 8 mm (Fig. 5b).

### Dental CBCT and micro-CT scanning

**Figure 6:**
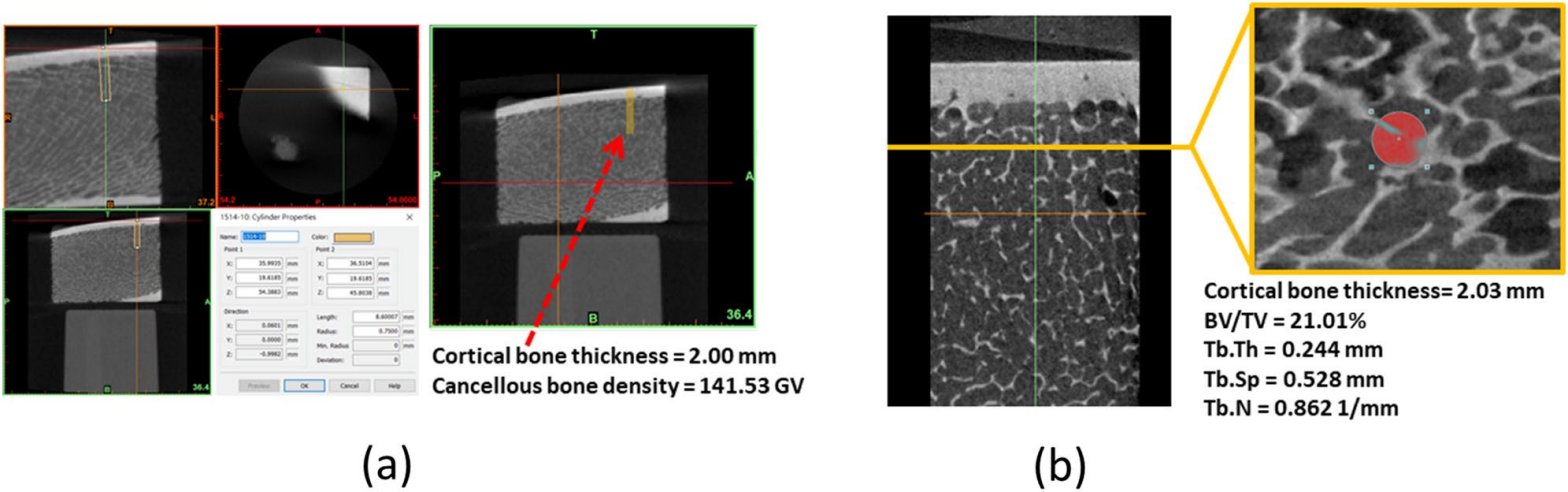
(**a**) Cortical bone thickness (in mm) and cancellous bone density (GV) measured from CBCT images; (**b**) cortical bone thickness (in mm) and four trabecular bone misconstruction parameters [BV/TV (%), Tb.Th. (mm), Tb.Sp. (mm), and Tb.N. (1/mm) measured from micro-CT images.

First, CBCT images of bone specimens were taken. The CBCT device used was a Promax 3D Max (Planmeca Oy, Finland), and the scanning parameters were a resolution of 200 µm, voltage of 96 kVp, and current of 12.5 mA; cancellous bone density was measured and expressed as a grayscale value (GV). The captured bone specimen DICOM images were imported into MIMICS 15.4 (Materialise, Leuven, Belgium) imaging software to measure the cortical bone thickness and cancellous bone density at the insertion position (region of interest [ROI]; Fig. 6a).

Subsequent scanning of bone specimens and orthodontic miniscrews was performed using micro-CT (SkyScan 2211, Bruker, Belgium), and the scanning parameters were a resolution of 25 μm, voltage of 110 kVp, and current of 300 μA. First, the micro-CT images of the bone specimens were imported into CTAn (Skyscan). Subsequently, the cortical bone thickness and the four trabecular bone microstructure parameters at the orthodontic miniscrew insertion positions (ROI) were measured. The four trabecular bone microstructure parameters measured are as follows: percent bone volume (bone volume/total volume; [BV/TV]), trabecular thickness (Tb.Th), trabecular number (Tb.N), and trabecular separation (Tb.Sp; Fig. 6b).

All parameters were measured by a radiologist with four years of experience in radiology. Measurement accuracy was validated before analyzing the seven parameters (cortical bone thickness in CBCT and micro-CT images, cancellous bone density in CBCT images, and four trabecular bone microstructure parameters). The intraclass correlation coefficient (ICC) was used to determine the reliability of the intraexaminer and interexaminer measurements. → To calculate the interexaminer error, the seven parameters from a certain inserted position of CBCT and micro-CT images were measured once each by two examiners and then compared. The ICC value was 0.899. To calculate the intraexaminer error, the seven parameters from a certain inserted position of CBCT and micro-CT images were measured two times by a single examiner, and those measurements were then compared. The ICC value was 0.955. These values indicated that the effect of intraexaminer and interexaminer error for this method was minimal and could be ignored in this study.

### Measurement of BMC% parameters

**Figure 7:**
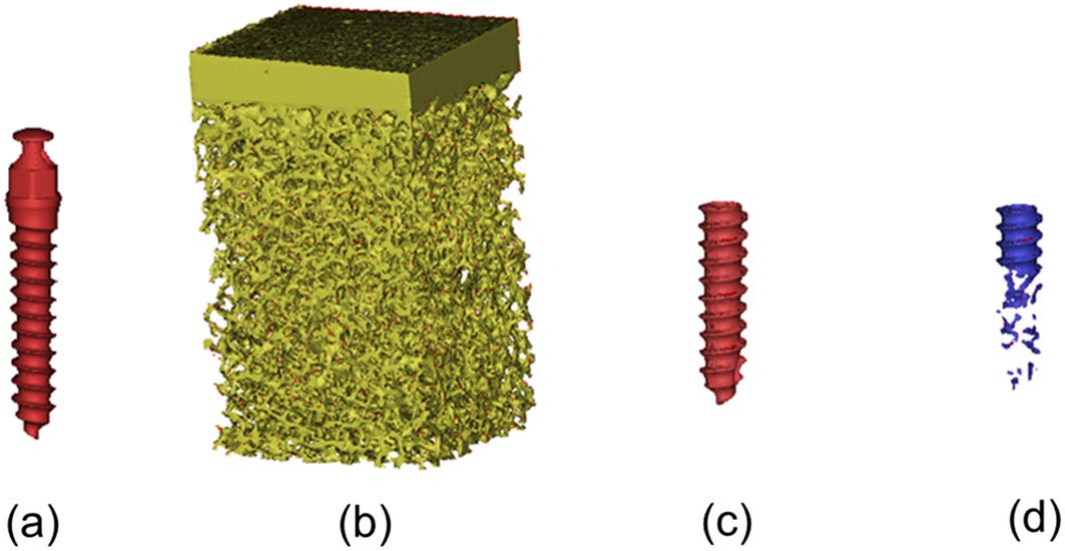
(**a**) 3D model of the orthodontic miniscrew, (**b**) 3D model of a small region of a bovine iliac bone specimen, (**c**) exterior surface of the orthodontic miniscrew inside a bone specimen (pBMCA), and (**d**) real bone–miniscrew contact area (BMCA).

The micro-CT images of the 12 bone specimens and the five dimensions of the orthodontic miniscrews were imported into MIMICS 15.4 for the construction of 3D models. In MIMICS, the 3D model of the orthodontic miniscrew (Fig. 7a) was moved to the designated miniscrew insertion sites of the 3D bone specimen model (Fig. 7b). With reference to the methods described in our previously published study^29^, the exterior surface of the orthodontic miniscrew inside a bone specimen (pBMCA) (Fig. 7c), the real bone to miniscrew contact area (BMCA) (Fig. 7d) and BMC% (BMCA divided by pBMCA) were calculated.

### Maximum ITV of orthodontic miniscrews

**Figure 8:**
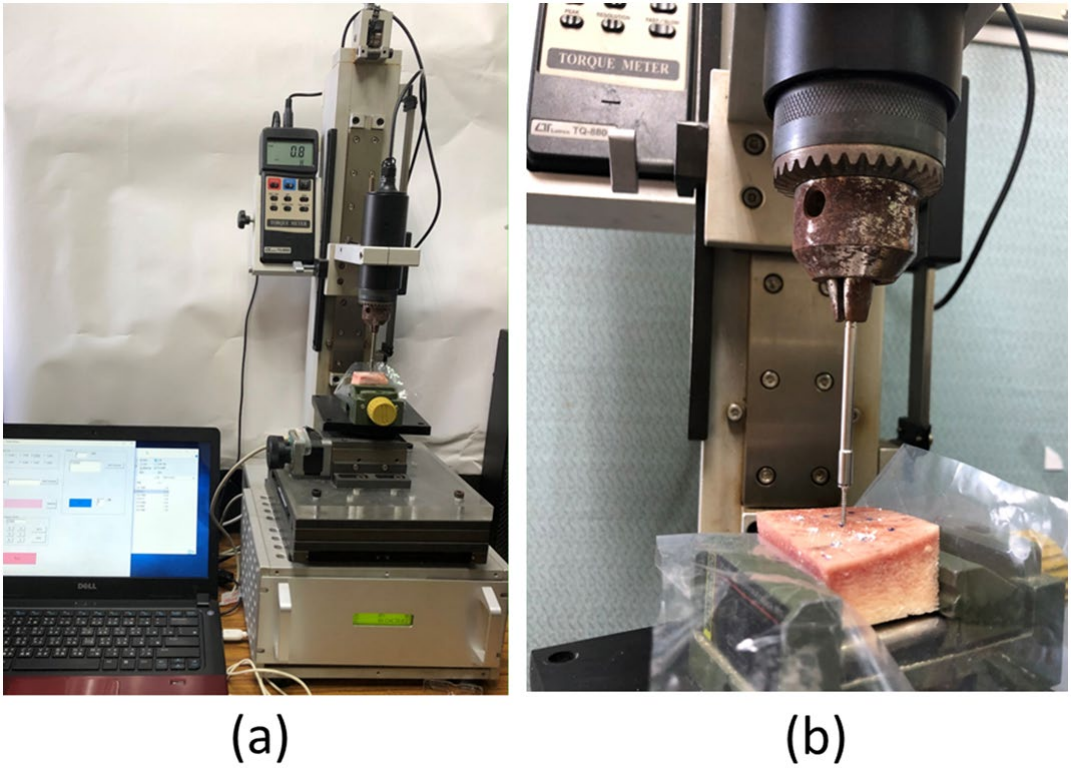
ITV measurement approach of ITV: (**a**) entire view, (**b**) close view.

A torque machine was used to measure the maximum ITV required to insert orthodontic miniscrews into the bone specimen. The torque load cell model of the torque machine used was TQ-8800 (Lutron Electronic Enterprise, Taipei, Taiwan) and the measurement resolution adopted was 0.1 N.cm. The experimental process complied with the provisions of the American Society for Testing and Materials (ASTM) F543; a normal force of 1.14 kg was applied, and the orthodontic miniscrews were inserted into the bone specimens (Fig. 8) under torsional force applied at a constant rate of 4 r/min.

### Statistical analysis

This experiment was divided into three parts. Herein, each part is discussed separately; then, the correlations between the three parts are discussed as follows:

1. Part I: Comparison of the influence of the dimensions of five different orthodontic miniscrews on the maximum ITV using a Kruskal–Wallis test and a post hoc Mann–Whitney U test.
2. Part II: Comparison between measurements of bone quality and bone quantity measured using CBCT and those measured using micro-CT images: paired *t* tests were used to compare the difference between cortical bone thickness measured using CBCT and that measured using micro-CT images; Pearson’s correlation coefficient was used to examine the correlations between the cancellous bone density (GV) measured by CBCT and the parameters of trabecular bone microstructure measured by micro-CT (BV/TV, Tb.Th, Tb.N, and Tb.Sp).
3. Part III: Comparison of the influence of the dimensions of five different miniscrews on BMC% through the use of a Kruskal–Wallis test and post hoc Mann–Whitney U test.
4. Part IV: Examination of the correlation between the maximum ITV and bone quality and quantity: Pearson’s correlation coefficient was used to examine the correlations between the maximum ITV of different orthodontic miniscrews, cortical bone thickness, cancellous bone density, and trabecular bone microstructure parameters.
5. Part V: Examination of the correlations between BMC% and bone quality and bone quantity: Pearson’s correlation coefficient was used to examine the correlations between BMC%, cortical bone thickness, cancellous bone density, and trabecular bone microstructure parameters.
6. Part VI: Exploration of the correlation between BMC% and the maximum ITV: Pearson’s correlation coefficient was used to explore the correlation between BMC% and the maximum ITV.

## Data Availability

The data that support the findings of this study are available from the corresponding author upon reasonable request.
